# Upstream Stimulatory Factors 1 and 2 Mediate the Transcription of Angiotensin II Binding and Inhibitory Protein*

**DOI:** 10.1074/jbc.M113.451054

**Published:** 2013-05-07

**Authors:** Miyuki Matsuda, Kouichi Tamura, Hiromichi Wakui, Akinobu Maeda, Masato Ohsawa, Tomohiko Kanaoka, Kengo Azushima, Kazushi Uneda, Sona Haku, Yuko Tsurumi-Ikeya, Yoshiyuki Toya, Yohei Maeshima, Akio Yamashita, Satoshi Umemura

**Affiliations:** From the ‡ Department of Medical Science and Cardiorenal Medicine, Yokohama City University Graduate School of Medicine, Yokohama 236-0004,; the § Department of Medicine and Clinical Science, Okayama University Graduate School of Medicine Dentistry and Pharmaceutical Sciences, Okayama 700-8558, and; the ¶ Department of Molecular Biology, Yokohama City University Graduate School of Medicine, Yokohama 236-0004, Japan

**Keywords:** Angiotensin, Gene Transcription, Receptors, Renin Angiotensin System, Transcription Regulation, Distal Tubule

## Abstract

The angiotensin II type 1 receptor (AT1R)-associated protein (ATRAP/Agtrap) promotes constitutive internalization of the AT1R so as to specifically inhibit the pathological activation of its downstream signaling yet preserve the base-line physiological signaling activity of the AT1R. Thus, tissue-specific regulation of Agtrap expression is relevant to the pathophysiology of cardiovascular and renal disease. However, the regulatory mechanism of *Agtrap* gene expression has not yet been fully elucidated. In this study, we show that the proximal promoter region from −150 to +72 of the mouse *Agtrap* promoter, which contains the X-box, E-box, and GC-box consensus motifs, is able to elicit substantial transcription of the *Agtrap* gene. Among these binding motifs, we showed that the E-box specifically binds upstream stimulatory factor (Usf) 1 and Usf2, which are known E-box-binding transcription factors. It is indicated that the E-box-Usf1/Usf2 binding regulates *Agtrap* expression because of the following: 1) mutation of the E-box to prevent Usf1/Usf2 binding reduces Agtrap promoter activity; 2) knockdown of Usf1 or Usf2 affects both endogenous Agtrap mRNA and Agtrap protein expression, and 3) the decrease in Agtrap mRNA expression in the afflicted kidney by unilateral ureteral obstruction is accompanied by changes in Usf1 and Usf2 mRNA. Furthermore, the results of siRNA transfection in mouse distal convoluted tubule cells and those of unilateral ureteral obstruction in the afflicted mouse kidney suggest that Usf1 decreases but Usf2 increases the *Agtrap* gene expression by binding to the E-box. The results also demonstrate a functional E-box-USF1/USF2 interaction in the human *AGTRAP* promoter, thereby suggesting that a strategy of modulating the E-box-USF1/USF2 binding has novel therapeutic potential.

## Teaser

**Background:** Regulation of angiotensin II type 1 receptor-interacting and inhibitory protein (ATRAP/Agtrap) is important in pathophysiology.

**Results:** Gene knockdown in cells and unilateral ureteral obstruction in mice indicate that Usf1 decreases and Usf2 increases Agtrap expression.

**Conclusion:** Interplay between E-box and Usf1/Usf2 is important for *Agtrap* regulation.

**Significance:** A strategy of modulating the E-box-Usf1/Usf2 interaction may have novel therapeutic potential.

## Introduction

Evidence has been accumulating that the activation of angiotensin II (Ang II)^2^ type 1 receptor (AT1R) through the tissue renin-angiotensin system plays a pivotal role in the pathogenesis of cardiovascular remodeling and renal injury (1, 2). The intrarenal activation of AT1R has also been proposed to play a role in the regulation of sodium and water reabsorption through constriction of the glomerular arteries, hence a direct effect on renal tubular transport function, and to evoke excessive sodium retention, resulting in hypertension, when thus inappropriately stimulated (3, 4). The C-terminal portion of the AT1R is involved in the control of AT1R internalization independent of G protein coupling, and it plays an important role in linking receptor-mediated signal transduction with the specific biological response to Ang II (5, 6). The AT1R-associated protein (ATRAP/Agtrap) was identified as an interacting molecule with the C-terminal domain of AT1R (7, 8), and previous *in vitro* and *in vivo* studies showed that Agtrap promotes constitutive internalization of the AT1R so as to specifically inhibit the pathological activation of its downstream signaling and yet preserve base-line physiological signaling activity (2, 9–17).

Although Agtrap is abundantly expressed in the renal nephron tubules, it is also widely expressed in many other cell types and tissues in addition to the kidney. Thus, it is important to elucidate the molecular mechanism of the cell type- and tissue-specific regulation of *Agtrap* gene expression to determine the regulatory machinery for the tissue Agtrap level and/or Agtrap activity under both physiological and pathological conditions. The balance of the endogenous expression of Agtrap and AT1R in local tissues is important for the regulation of tissue AT1R signaling. Down-regulation of Agtrap and/or up-regulation of AT1R at local tissue sites together with the resultant pathological activation of the tissue renin-angiotensin system are pathogenetic mechanisms that may be responsible for cardiovascular and renal disease. For example, in Ang II-infused mice and genetically hypertensive rats, the development of hypertension and organ injury, such as cardiac hypertrophy and renal fibrosis, was reportedly accompanied by a decrease in the tissue Agtrap expression without altered AT1R expression (2, 15–19). In addition, we previously showed that serum starvation stimulates *Agtrap* gene expression in mouse distal convoluted tubule cells (mDCT cells) and that Runx3, one of the Runt-related transcription factors, is involved in the transcriptional activation of *Agtrap* gene expression (20). However, the regulatory mechanism of *Agtrap* gene expression in relation to organ injury needs further investigation to elucidate the relationship of the regulation of Agtrap expression with the pathophysiology of cardiovascular and renal disease at the molecular level.

The transcription factors upstream stimulatory factor (USF/Usf) 1 and USF2/Usf2 were originally identified in HeLa cells by biochemical analysis (21, 22). The human cDNA cloning of USF1 and USF2 revealed that the USFs belong to the c-Myc-related family of DNA-binding proteins, which have a helix-loop-helix motif and a leucine repeat, and that USF interacts with its target DNA as a dimer (23). Previous examination of the tissue and cell type distribution of USF1 and USF2 revealed that although both are ubiquitously expressed, different ratios of USF homo- and heterodimers are found in different tissues and cell types (24). The results of mouse Usf1 cDNA cloning showed a high level of sequence homology between the mouse and human USF1 genes (25). Previous studies that were undertaken to assign a physiological role to the Usfs *in vivo*, including the disruption of Usf1 and Usf2 genes in mice, revealed that Usf1 and Usf2 play a role in the modulation of glucose and lipid metabolism by modulation of their trans-activating efficiency (26–29). Subsequent studies also showed that Usf1 and Usf2 are involved in the pathophysiology of several metabolic disorders, including familial hypercholesterolemia and diabetic nephropathy (30–33). In this study, we show that the proximal promoter region (−72 to −43) of the mouse *Agtrap* gene contains an “E-box (CANNTG)” sequence, which is a putative binding site for Usf1 and Usf2 that interacts with these transcription factors. It is shown both *in vitro* and *in vivo* that Usf1 decreases and Usf2 increases the *Agtrap* gene expression through their binding to the E-box.

## EXPERIMENTAL PROCEDURES

### 

#### 

##### Cell Culture

The mDCT cells were kindly provided by Dr. Peter A. Friedman (University of Pittsburgh School of Medicine). These cells have been shown to have a phenotype of a polarized tight junction epithelium along with both morphological and functional features retained from the parental cells (14, 34–36). The mDCT cells also express the endogenous AT1R and Agtrap (14). Human embryonic kidney-derived 293 (HEK293) cells were cultured according to the American Type Culture Collection (ATCC) protocol, as described previously (37, 38).

##### Animals and Treatment

Adult C57BL/6 mice were purchased from Oriental Yeast Kogyo (Tokyo, Japan). The procedure of unilateral ureteral obstruction (UUO) was performed using C57BL/6 mice, as described previously (20, 39). Briefly, with the mice under anesthesia, the left ureter was ligated with 4-0 silk at two locations and then cut between the ligatures to prevent retrograde urinary tract infection. Mice that were operated on were sacrificed under anesthesia 7 days after UUO. Sham operation was also performed in which the ureters were manipulated but not ligated. Seven days after the sham operation, mice were sacrificed to obtain control kidneys. The procedures were performed in accordance with the National Institutes of Health guidelines for the use of experimental animals. All of the animal studies were reviewed and approved by the Animal Studies Committee of Yokohama City University.

##### Plasmid Construction and Transcriptional Mouse Agtrap and Human AGTRAP Promoter Assay

For the analysis of the mouse *Agtrap* promoter, 5022-, 2943-, 2090-, 1272-, 972-, 613-, 453-, 374-, and 222-bp mouse *Agtrap* promoter fragments (−4950, −2871, −2018, −1200, −900, −541, −381, −302, and −150 to +72 of the putative transcriptional start site, respectively) were amplified from C57BL/6J genomic DNA, using the pair of primers indicated in Table 1, and then subcloned into the multicloning sites of pBluescript. A 613-bp *Agtrap* promoter fragment (−541 to +72 of the putative transcriptional start site)-containing plasmid was used as a template to construct mutations in the X-box, E-box, and GC-box by oligonucleotide (ODN)-directed mutagenesis (40–42). The sequences of the oligonucleotide used to create the mutated X-box (X-box mt), mutated E-box (E-box mt), mutated GC-box (GC-box mt, and mutated X- and E-boxes (X/E-box mt) are also shown in Table 1. To normalize transfection efficiency, we employed the Dual-Luciferase Assay System (Promega) for the transcriptional *Agtrap* promoter assay using pGL3-basic plasmid-based luciferase constructs, as described previously (20, 36).

For analysis of the human *AGTRAP* promoter, 575-bp *AGTRAP* promoter fragments (−480 to +95 of the putative transcriptional start site, NC_000001.9) containing two adjacent wild-type or mutated E-box motifs, were gene-synthesized (Eurofins MWG Operon). The human *AGTRAP* promoter assay using the Dual-Luciferase Assay System (Promega) was performed using pGL3- and pGL4.1-basic plasmid-based luciferase constructs (20, 36).

##### Real Time Quantitative RT-PCR Analysis

Total RNA was extracted and purified using the RNeasy kit (Qiagen), and the cDNA was synthesized using SuperScript VILO (Invitrogen). Real time quantitative RT-PCR was performed by incubating the RT product with the TaqMan Universal PCR Master Mix and designed TaqMan FAM^TM^ dye-labeled probes for Usf1, Usf2, and Agtrap (Applied Biosystems), and a TaqMan VIC dye-labeled probe as the internal control (18 S rRNA Endogenous Control, Applied Biosystems) in the same reaction mixture (CFX96 system, BIO-RAD), essentially as described previously (20).

##### Immunoblot Analysis

A 14-amino acid synthetic peptide corresponding to amino acids 148–161 of the C-terminal tail of mouse (DBA/2J) ATRAP was used for the generation of a polyclonal anti-ATRAP antibody (7), and the characterization and specificity of the anti-ATRAP antibody were described previously (9, 15, 43). Antibodies for USF1 (C-20 sc-229, Santa Cruz Biotechnology), USF2 (ab32616, Abcam), TATA-binding protein (ab818[1TBP18], Abcam), and α-tubulin (ab40742 Abcam) were also used. Immunoblot analysis was performed as described previously (9, 15, 43), and the images were analyzed using a FUJI LAS3000mini Image Analyzer (FUJI Film, Tokyo, Japan).

##### Electrophoretic Mobility Shift Assay (EMSA)

Nuclear extracts from mDCT cells (70–80% confluent, a 15-cm diameter dish) were prepared with a modification of the protocols of Dignam *et al.* (44) and Swick *et al.* (45). The final protein concentration was adjusted to 1 mg/ml. EMSA was performed essentially as described previously (46, 47). Briefly, single-stranded ODN sequences were biotin-labeled at 3′-ends by the manufacturer, annealed to each other, and used as the probe. The ODN sequences for the E-box and mutated E-box (E-box mt) are shown in Table 1. Nuclear extracts (2 μg) were incubated on ice in a 20-μl EMSA binding reaction mixture containing 10 mm Tris-HCl, pH 7.5, 50 mm NaCl, 1 mm EDTA, pH 8.0, 4% glycerol, 1 μg of BSA, and 1 μg of double-stranded poly(dI-dC) in the presence or absence of a specific double-stranded competitor DNA and biotin-labeled DNA probe. The incubation mixture was loaded onto a 5% polyacrylamide mini (7.5 × 9.0 cm) gel in 0.5× TBE and electrophoresed at 350 V for 25 min, followed by transfer of DNA from the gel onto nylon membranes (Hybond-N+, GE) by cross-linking the transferred DNA to the membrane and rinsing with the TN buffer (100 mm Tris-HCl, pH 7.5, 150 mm NaCl). After blocking the incubation with Blocking Reagent (FP1020, PerkinElmer Life Sciences), incubating with streptavidin-horseradish peroxidase (HRP) conjugate (NEL750, PerkinElmer Life Sciences), and washing to remove unreacted excess reagent with PBST (0.05% Tween 20/PBS), the biotin-labeled DNA was visualized by chemiluminescence (Immobilon Western Detection Reagent, Millipore) and analyzed using an LAS3000mini Image Analyzer (FUJI Film, Japan).

##### Streptavidin-Biotin Complex Assay

Streptavidin-biotin complex assay was performed using 3′-biotin-labeled oligonucleotides corresponding to the *Agtrap* E-box and X-box (Table 1), essentially as described previously (28, 48, 49). The streptavidin that was immobilized on agarose CL-4B (85881, Sigma) was pretreated with TN buffer containing 1% BSA and incubated with 50 μg of nuclear extracts from mDCT cells on ice in a 200-μl EMSA binding buffer for 20 min. After five washing steps with EMSA binding buffer, the streptavidin-biotin-DNA complex was eluted with SDS buffer, and a one-fifth volume was used for immunoblot analysis.

##### Chromatin Immunoprecipitation (ChIP) Assay

ChIP assay was performed essentially according to the manufacturer's protocol (Active Motif) (50, 51). Briefly, mDCT or HEK293 cells were treated with formalin to cross-link the protein-DNA complexes, and glycine was added to stop the reaction. The cells were lysed with 300 μl of lysis buffer (50 mm Tris-HCl, pH 8.0, 10 mm EDTA, pH 8.0, 1% SDS, protease inhibitor mixture; P8340, Sigma), and the lysates were sonicated using the Bioruptor Sonication System (250 watts, 30 s on and 30 s off/30 cycle; Bioruptor UCD-250, COSMO BIO, Tokyo, Japan) to reduce the DNA fragments. Subsequently, the sonicated lysates were divided into three equal aliquots for immunoprecipitation with specific antibodies, immunoprecipitation with control IgG (rabbit anti-HA antibody; 561, MBL, Japan), and input reference. After immunoprecipitation with an anti-USF1 antibody (C-20 sc-229, Santa Cruz Biotechnology), anti-USF2 antibody (C-20 sc-862, Santa Cruz Biotechnology), anti-SREBP1 antibody (H-160 sc-8984, Santa Cruz Biotechnology), anti-BMAL1 antibody (ab3350, Abcam), or control IgG, DNA was purified from the antibody-bound and unbound input fractions. The anti-USF1 antibody and anti-USF2 antibody used in the ChIP assay were characterized in detail in a previous study (28). Enrichment of the mouse *Agtrap* promoter sequences in the respective bound fractions was estimated by quantitative PCR with the SsoFast EvaGreen system (Bio-Rad) using the primers shown in Table 1 to detect the 134-bp fragment (−65 to +69 of the transcriptional start site).

For the ChIP analysis of human *AGTRAP*, HEK293 cells were treated with formalin to cross-link the protein-DNA complexes, and then the cells were lysed with lysis buffer and sonicated to reduce the DNA fragments. After immunoprecipitation with an anti-USF1 antibody, anti-USF2 antibody, anti-SREBP1 antibody, anti-BMAL1 antibody or control IgG, DNA was purified from the antibody-bound and unbound input fractions. Enrichment of the *AGTRAP* promoter or exon3 sequences in the bound fractions was estimated by quantitative PCR using the primers in Table 1 to detect 161- or 102-bp fragments, respectively. The target proteins in the co-immunoprecipitates were also subjected to immunoblot analysis and were visualized by TrueBlot (Affymetrix).

##### Statistical Analysis

All the quantitative data are expressed as the means ± S.E. For comparisons between groups, Student's *t* test was employed. Differences were considered to be statistically significant at *p* < 0.05.

## RESULTS

### 

#### 

##### Determination of the Minimal Mouse Agtrap Promoter

**FIGURE 1.**
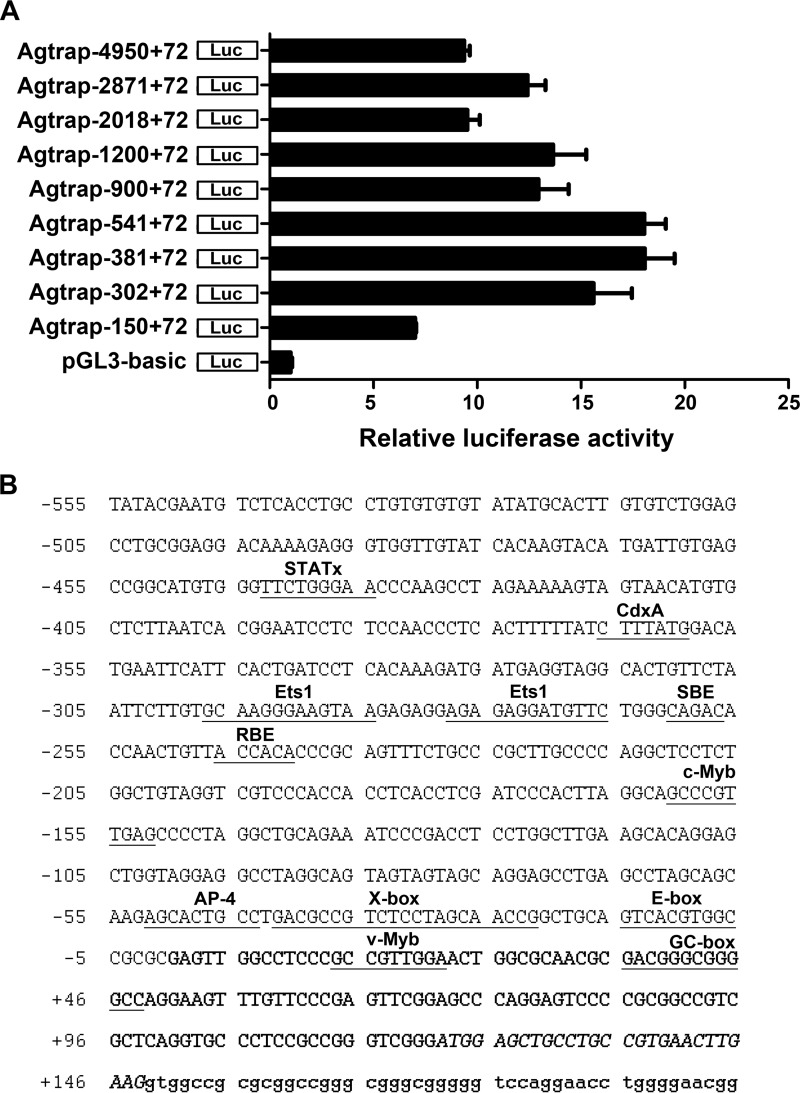
**Identification of mouse *Agtrap* promoter region.** *A*, functional analysis of the mouse *Agtrap* promoter in mDCT cells. The *Agtrap* promoter-luciferase constructs were transiently transfected into mDCT cells, and luciferase assay was performed. The relative luciferase activities were calculated relative to those achieved with the promoterless control plasmid (pGL3-basic). Data are expressed as the means ± S.E. (*n* = 4). *B*, nucleotide sequence of the mouse *Agtrap* promoter region and putative transcription factor-binding motifs. The nucleotides are numbered at the *left* with the putative initiation site of transcription designated as +1. The untranslated and translated nucleotides of exon 1 are designated by the *bold letters* and the *bold italic letters*, respectively. The nucleotides in a portion of intron 1 are indicated by the *small letters*.

To determine the minimal region required for basal activity of the core promoter of the *Agtrap* gene, the 5-kb promoter region upstream of its transcriptional start site was isolated. Then, we generated a series of luciferase reporter plasmids containing the various *Agtrap* proximal promoter regions, which are illustrated in Fig. 1*A*. To determinate the minimal *Agtrap* promoter, we transfected these plasmids into mDCT cells, and luciferase activity was measured. Although the luciferase activity was gradually increased by the deletion from −4950 to −541, further deletion, *i.e.* from −381 to −150, resulted in a decrease in the luciferase activity of the *Agtrap* reporter constructs (Fig. 1*A*). Consistent with this finding, this region contains two important *Agtrap* regulatory elements, the SMAD-binding element (−261 to −257) and the Runt-binding element (−246 to −241) (20). Intriguingly, the promoter region from −150 to +72 maintained the luciferase activity of the *Agtrap* reporter constructs. This suggested that this region contains important regulatory elements for *Agtrap* gene transcription. To identify the candidate transcription factors involved in *Agtrap* gene transcription, we next performed a computational sequence analysis of the *Agtrap* proximal promoter region using TFSEARCH: Searching Transcription Factor-binding Sites software and identified the consensus binding motifs for several transcription factors (Fig. 1*B*).

##### Functional Involvement of X-box, E-box, and GC-box in the Proximal Mouse Agtrap Promoter Activity

**FIGURE 2.**
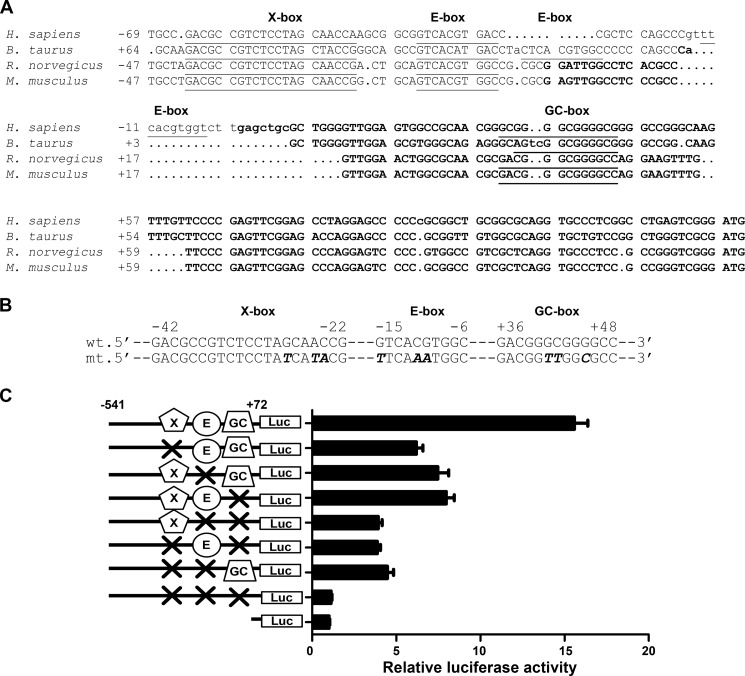
**Involvement of the X-box, E-box, and GC-box regions in the transcriptional activation of the mouse *Agtrap* promoter in mDCT cells.** *A*, alignment of the proximal regions of the human (*Homo sapiens*), cow (*Bos taurus*), rat (*Rattus norvegicus*), and mouse (*Mus musculus*) *Agtrap* genes. The nucleotides are numbered at the *left* with the putative initiation site of transcription designated as +1. The putative transcription factor binding motifs are indicated with *underlines. B*, construction of site-directed mutations in the X-box, E-box, and GC-box in the mouse *Agtrap* promoter sequence. Wild-type sequences (*wt*) and mutated sequences (*mt*) are shown. *C*, effects of mutations in the X-box, E-box, and GC-box on the transcriptional activity of the mouse *Agtrap* promoter (−541 to +72 of the transcriptional start site)-luciferase hybrid gene in mDCT cells. The relative luciferase activities were calculated relative to those achieved with the promoterless control plasmid. Data are expressed as the means ± S.E. (*n* = 4).

Among the consensus binding motifs of the transcription factors listed in Fig. 1*B*, there are highly homologous sequences of the X-box (5′-GTCCCTAGCAAC-3′) (52), E-box (5′-CATGTG-3′ or 5′-CANNTG-3′), and GC-box (5′-GGAGGGGGG(A/C)GG-3′) (53), which are highly conserved in mammals (Fig. 2*A*). To examine the functional role of these conserved elements in the regulation of *Agtrap* gene transcription, we mutated the core binding sequences of the X-box, E-box, and GC-box in the *Agtrap* promoter, X-box mt, E-box mt, and GC-box mt (Fig. 2*B*). Although the promoter region from −541 to +72 of the putative transcriptional start site of the *Agtrap* gene exhibited substantial luciferase activity in mDCT cells, site-directed mutations of the X-box, E-box, or GC-box decreased the luciferase activity to 39.7 ± 2.5% (X-box mt), 48.2 ± 4.1% (E-box mt), and 51.2 ± 3.0% (GC-box mt) of that achieved with the wild-type promoter, respectively (Fig. 2*C*). Mutations of any two of the three consensus motifs further decreased the luciferase activity (E-box/GC-box mt, 25.3 ± 1.4%; X-box/GC-box mt, 24.7 ± 1.3%; X-box/E-box mt, 28.6 ± 2.4%) relative to that achieved with the wild-type promoter, whereas mutation of all three motifs reduced the luciferase activity almost to the background reference level (X-box/E-box/GC-box mt, 7.2 ± 0.3%). These results indicate that the three binding motifs of the X-box, E-box, and GC-box are important for the basal transcriptional activity directed by the minimal *Agtrap* promoter and suggest that these binding motifs independently modulate the promoter activity of the *Agtrap* gene.

##### Identification of the E-box as a Transcription Factor-binding Site in the Mouse Agtrap Promoter

**FIGURE 3.**
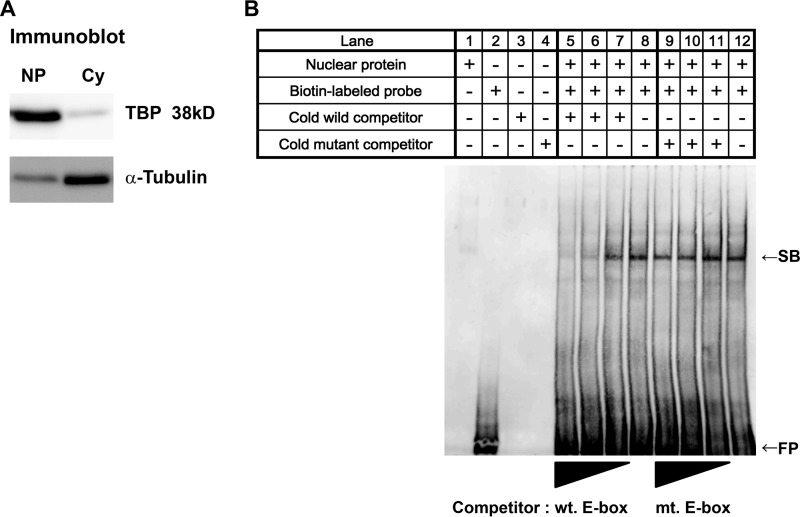
**Identification of nuclear factors binding to the E-box (−72 to −43) of the mouse *Agtrap* promoter by EMSA.** *A*, immunoblot analysis shows TATA-binding protein (*TBP*) and α-tubulin in the nuclear extract (*NP*) and cytosolic extract (*Cy*), respectively. *B*, electrophoretic mobility shift and competition analyses of complexes formed by nuclear factors with the E-box (−72 to −43) of the *Agtrap* promoter. The E-box ODNs were biotin-labeled at the 3′-end and used as the labeled probe. Nuclear extracts (2 μg) from mDCT cells were incubated with the probe. In electrophoretic shift competition assay, 50, 12.5, and 2.5 pmol of unlabeled wild-type E-box (*wt*) or mutated E-box (*mt*) ODNs were added to the reaction mixture. *SB* indicates a shifted band derived from specific DNA-protein complexes. *FP,* free probes.

Among the X-box, E-box, and GC-box in the *Agtrap* proximal promoter, the canonical E-box is a target for many genes involved in pathophysiological conditions such as diabetic nephropathy and fibrotic disease (33, 54, 55). Therefore, we focused on the functional characterization of the E-box in the regulation of the *Agtrap* promoter. To determine whether the E-box is capable of binding transcription factors, nuclear extracts were prepared from mDCT cells (Fig. 3*A*), and EMSA analysis was performed with an Agtrap promoter fragment (−72 to −43) probe containing the E-box but not the X-box or GC-box (Table 1). The E-box probe formed a DNA-protein complex (Fig. 3*B*, *lanes 8* and *12*), and the formation of the complex was completely impaired by the addition of an excess amount of the unlabeled probe with a wild-type sequence (Fig. 3*B*, *lanes 5–7*), but not by a mutated probe (Fig. 3*B*, *lanes 9–11*). These results indicate that there are nuclear factors that bind to the E-box sequence of the *Agtrap* promoter.

**TABLE 1:** **Primer sequences used in the study**

Primers		Primer sequences
**Construction of wild-type and mutated *Agtrap* promoter-containing plasmids**		
−4950 to −353	Forward	5′GCCATTCCCTGAGCTGTTGAGGGCCCTTCACTGAAAGGCTTCTTGGT3′
	Reverse	5′CTTTGTGAGGATCAGTGAATGAATTCATGTCCATAAAGATAAAAAGTGA3′
−2871 to 353	Forward	5′CTAGAGAAGGTACCCAAGGAGCTAAACGGATCTGCAACCCTATAG3′
	Reverse	5′CTTTGTGAGGATCAGTGAATGAATTCATGTCCATAAAGATAAAAAGTGA3′
−2018 to −353	Forward	5′GCTATGTGGTTAAAGGCACTTGCCACACCAGCCTGTCGACTGGCC3′
	Reverse	5′CTTTGTGAGGATCAGTGAATGAATTCATGTCCATAAAGATAAAAAGTGA3′
−1200 to +72	Forward	5′ggggtaCCAACTTTTGCTATGTTGGGCAAGTGGACTCCA3′
	Reverse	5′cgggatccGAACTCGGGAACAAACTTCCT3′
−900 to +72	Forward	5′ggggtaCCCCTTTCTTGACTTCAGGTCCTGTCTCCCTTTCC3′
	Reverse	5′cgggatccGAACTCGGGAACAAACTTCCT3′
−541 to +72	Forward	5′gcggtACCTGCCTGTGTGTGTATATGCACTT3′
	Reverse	5′cgggatccGAACTCGGGAACAAACTTCCT3′
−381 to +72	Forward	5′gcggtACCCTCACTTTTTATCTTTATGG3′
	Reverse	5′cgggatccGAACTCGGGAACAAACTTCCT3′
−302 to +72	Forward	5′ggggtacCTTGTGCAAGGGAAGTAAGA3′
	Reverse	5′cgggatccGAACTCGGGAACAAACTTCCT3′
−150 to +72	Forward	5′gcggtaCCCTAGGCTGCAGAAATCCC3′
	Reverse	5′cgggatccGAACTCGGGAACAAACTTCCT3′

**Construction of mutated *Agtrap* promoter-containing plasmids**		
X-box-mt	Forward	5′CTGCCTGACGCCGTCTCCTATCATACGGCTGCAGTCACGTGGCCG3′
	Reverse	5′CGGCCACGTGACTGCAGCCGTATGATAGGAGACGGCGTCAGGCA3′
E-box-mt	Forward	5′TCCTAGCAACCGGCTGCATTCAAATGGCCGCGCGAGTTGGCCT3′
	Reverse	5′AGGCCAACTCGCGCGGCCATTTGAATGCAGCCGGTTGCTAGGA3′
GC-box-mt	Forward	5′GAACTGGCGCAACGCGACGGTTGGCGCCAGGAAGTTTGTTCCCGA3′
	Reverse	5′TCGGGAACAAACTTCCTGGCGCCAACCGTCGCGTTGCGCCAGTTC3′
X/E-box-mt	Forward	5′TCCTATCATACGGCTGCATTCAAATGGCCGCGCGAGTTGGCCT3′
	Reverse	5′AGGCCAACTCGCGCGGCCATTTGAATGCAGCCGTATGATAGGA3′

**Electrophoretic mobility shift assay (EMSA) and oligonucleotide precipitation assay**		
E-box ODN		5′CCGGCTGCAGTCACGTGGCCGCGCGAGTTG3′ and 5′CAACTCGCGCGGCCACGTGACTGCAGCCGG3′
E-box-mt ODN		5′CCGGCTGCATTCAAATGGCCGCGCGAGTTG3′ and 5′CAACTCGCGCGGCCATTTGAATGCAGCCGG3′
X-box ODN		5′CTGCCTGACGCCGTCTCCTAGCAACCGGCTG3′ and 5′CAGCCGGTTGCTAGGAGACGGCGTCAGGCAG3′

**Chromatin immunoprecipitation (ChIP) assay**		
Mouse *Agtrap* promoter	Forward	5′CCTAGCAGCAAGAGCAGCT3′
	Reverse	5′GAACTCGGGAACAAACTTCCT3′
Human *AGTRAP* promoter	Forward	5′ACAGTCCGCTTCCTGGAATA3′
	Reverse	5′GCCGCTTGGTTGCTAGGAGACGGCGTCGGCAGC3′
Human *AGTRAP* exon 3	Forward	5′GGCTGCATTGTATTCTCAGG3′
	Reverse	5′CTTATGGCGTCGATGGAGTC3′

##### Specific Binding of Usf1 and Usf2 to the E-box of the Mouse Agtrap Promoter

**FIGURE 4.**
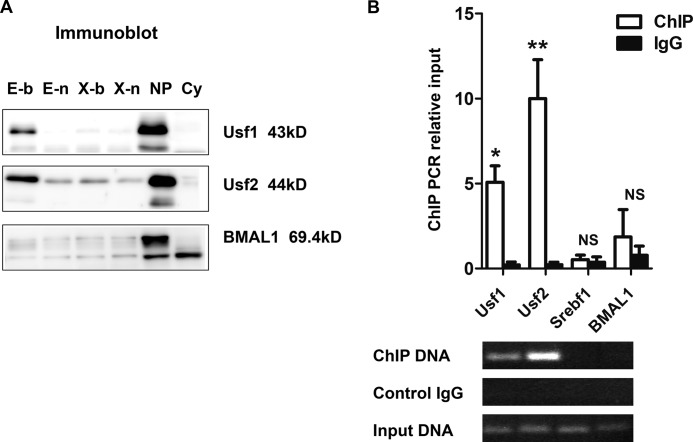
**Identification of Usf1 and Usf2 interaction with the E-box (−72 to −43) of the mouse *Agtrap* promoter by streptavidin-biotin complex assay and ChIP assay.** *A*, streptavidin-biotin complex assay. Nuclear extracts from mDCT cells were incubated with streptavidin immobilized on agarose beads. The streptavidin-biotin-DNA complex was eluted with SDS buffer and visualized by immunoblot analysis. *E-b,* E-box biotin-labeled probe; *E-n,* E-box nonlabeled probe; *X-b,* X-box biotin-labeled probe; *X-n,* X-box nonlabeled probe; *NP,* nuclear extracts; *Cy,* cytosolic extracts. *B*, ChIP assay. The mDCT cells were treated with an anti-USF1 antibody, anti-USF2 antibody, anti-SREBP1 antibody, anti-BMAL1 antibody, or control IgG (rabbit anti-HA antibody). Co-immunoprecipitated DNA was purified and estimated by quantitative PCR. In the *upper panel* the relative amount of DNA fragment detected per antibody is shown. In the *lower panel*, the quantitative PCR products, which were loaded on 3% agarose gels and visualized by ethidium bromide staining, are shown. Experiments were independently repeated at least three times, and data are expressed as the means ± S.E. *, *p* < 0.05; **, *p* < 0.01, *versus* control IgG. *NS,* not significant.

Several candidate transcription factors, including Usf1, Usf2, BMAL1/Arnt1, and Srebf1, are reported to be capable of binding to the E-box sequence. Among these factors, Usf1, Usf2, and BMAL1/Arnt1, but not Srebf1 mRNA, were detectably expressed on RT-PCR and immunoblot analyses in mDCT cells (data not shown). We then examined whether Usf1, Usf2, and/or BMAL1 interact with the E-box of the *Agtrap* promoter using a biotin-labeled E-box probe and X-box probe. These biotin-labeled probes were individually mixed with the nuclear extracts of mDCT cells and pulled down using streptavidin-agarose. The results showed that substantial amounts of Usf1 (43 kDa) and Usf2 (44 kDa) proteins from nuclear extracts were pulled down with the biotin-labeled E-box, but not the X-box, of the *Agtrap* promoter (Fig. 4*A*). However, no binding of BMAL1 to the biotin-labeled E-box or X-box in the *Agtrap* promoter was observed.

We next performed ChIP analysis to determine whether Usf1 and Usf2 physiologically interacted with the *Agtrap* promoter region. As shown in Fig. 4*B*, the 134-bp E-box containing the sequence from −65 to +69 of the transcriptional start site of the *Agtrap* promoter was recovered from mDCT cells after immunoprecipitation of sheared genomic DNA with an anti-USF1 antibody and anti-USF2 antibody but not after immunoprecipitation with an anti-SREBP1 antibody or anti-BMAL1 antibody. Quantitative PCR analysis confirmed that Usf1 and Usf2 are present in the *Agtrap* E-box promoter region, and the corresponding genomic DNA was enriched with both an anti-USF1 antibody (*, *p* < 0.05, *versus* IgG control) and anti-USF2 antibody (**, *p* < 0.01, *versus* IgG control) but not with an anti-SREBP1 antibody or anti-BMAL1 antibody. These data provide evidence for the occupancy by Usf1 and Usf2, but not Srebf1 or BMAL1, of the mouse *Agtrap* promoter E-box *in vivo*.

##### Functional Involvement of Usf1 and Usf2 in Mouse Agtrap Promoter Activity

**FIGURE 5.**
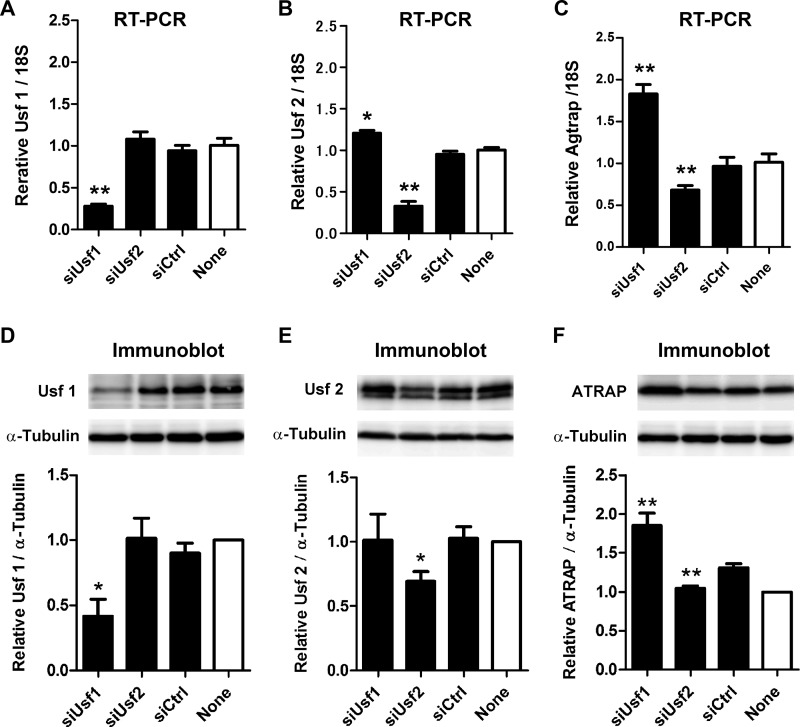
**Effects of specific knockdown of Usf1 and Usf2 by small interference (si)RNA on endogenous *Agtrap* gene expression in mDCT cells.** *A–C,* quantitative RT-PCR analysis showing the effects of respective siRNA transfection on the relative Usf1 (*A*), Usf2 (*B*), and Agtrap (*C*) mRNA levels. RNA quantity was normalized to the signal generated by constitutively expressed 18 S rRNA and is expressed relative to that achieved with extracts derived from nontreated mDCT cells (none). Experiments were independently repeated at least three times, and the data are expressed as the means ± S.E. *, *p* < 0.05; **, *p* < 0.01, *versus* control siCtrl. *D–F*, immunoblot analysis showing the effects of the respective siRNA transfection on the relative Usf1 (*D*), Usf2 (*E*), and Agtrap (*F*) protein levels. Representative immunoblots are shown, and protein expression levels are expressed relative to those achieved with extracts derived from nontreated mDCT cells (*none*). Experiments were independently repeated at least three times, and data are expressed as the means ± S.E. *, *p* < 0.05; **, *p* < 0.01, *versus* control siCtrl.

To determine whether Usf1 and Usf2 are involved in the transcriptional regulation of the *Agtrap* gene in mDCT cells, we examined the effect of Usf1 and Usf2 siRNAs transfection on endogenous *Agtrap* gene expression. The mRNA and protein levels of Usf1 (Fig. 5, *A* and *D*) and Usf2 (Fig. 5, *B* and *E*) were significantly decreased after transfection with their respective siRNA. In addition, although the Usf2 mRNA level was slightly increased by Usf1 knockdown (Fig. 5*B*), the Usf2 protein level was not affected (Fig. 5*E*). Intriguingly, although the siRNA reduction of Usf1 resulted in a significant increase in the levels of the Agtrap mRNA (Fig. 5*C*, *p* < 0.01, siUsf1 *versus* siCtrl) and protein (Fig. 5*F, p* < 0.01, siUsf1 *versus* siCtrl), Usf2 knockdown significantly decreased the Agtrap mRNA (Fig. 5*C, p* < 0.01, siUsf2 *versus* siCtrl) and protein (Fig. 5*F*, *p* < 0.01, siUsf1 *versus* siCtrl). These results show that Usf1 and Usf2 exert negative and positive regulatory effects on *Agtrap* gene expression, respectively.

##### Pathophysiological Relevance of Usf1 and Usf2 in Mouse Agtrap Gene Expression in the Kidney

**FIGURE 6.**
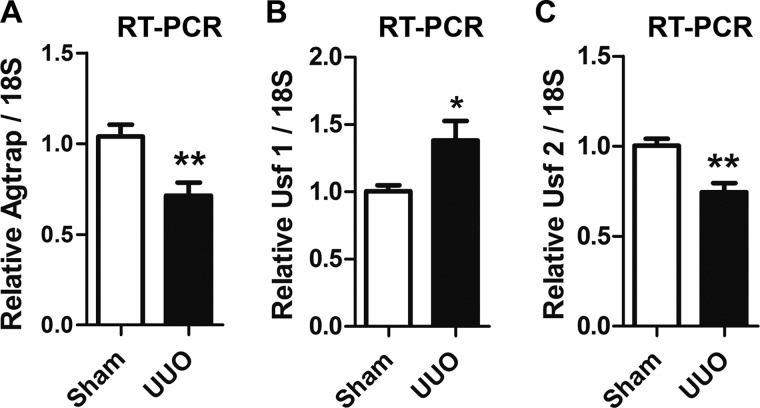
**Regulation of Usf1, Usf2, and Agtrap mRNA in the affected kidney by UUO.** *A–C,* quantitative RT-PCR analysis showing the effects of UUO on the relative Agtrap (*A*), Usf1 (*B*), and Usf2 (*C*) mRNA levels. RNA quantity was normalized to the signal generated by the constitutively expressed 18 S rRNA and is expressed relative to the level achieved with extracts derived from sham-operated kidney (*n* = 6). *, *p* < 0.05; **, *p* < 0.01, *versus* sham. Data are expressed as the means ± S.E.

To understand the pathophysiological roles of Agtrap in target organ injury, it is necessary to investigate the regulation of the expression of the *Agtrap* gene in response to pathological stimuli. UUO is a well established experimental model of progressive tubulo-interstitial fibrosis. UUO leads to changes in renal hemodynamics, inflammatory responses in the kidney, tubular hypertrophy, and interstitial fibrosis of the affected kidney by stimulating the renin-angiotensin system (39). Since we previously showed that the Agtrap mRNA level was suppressed in the affected kidney by UUO (20), we examined whether the change in *Agtrap* gene expression is accompanied by any modulation of the *Usf1* or *Usf2* gene expression in the UUO kidney. According to the results of quantitative RT-PCR analysis, while the Usf1 mRNA expression was significantly up-regulated in the affected kidney after 7 days of UUO (Fig. 6*B*), the Usf2 mRNA expression was significantly down-regulated in the affected kidney by UUO (Fig. 6*C*), with a concomitant decrease in the Agtrap mRNA expression (Fig. 6*A*). These results *in vivo* are consistent with the notion that Usf1 and Usf2 are inhibitory and stimulatory transcription factors for the *Agtrap* gene, respectively.

##### Functional Involvement of the Two Adjacently Located E-box Motifs in Proximal Human AGTRAP Promoter Activity

**FIGURE 7.**
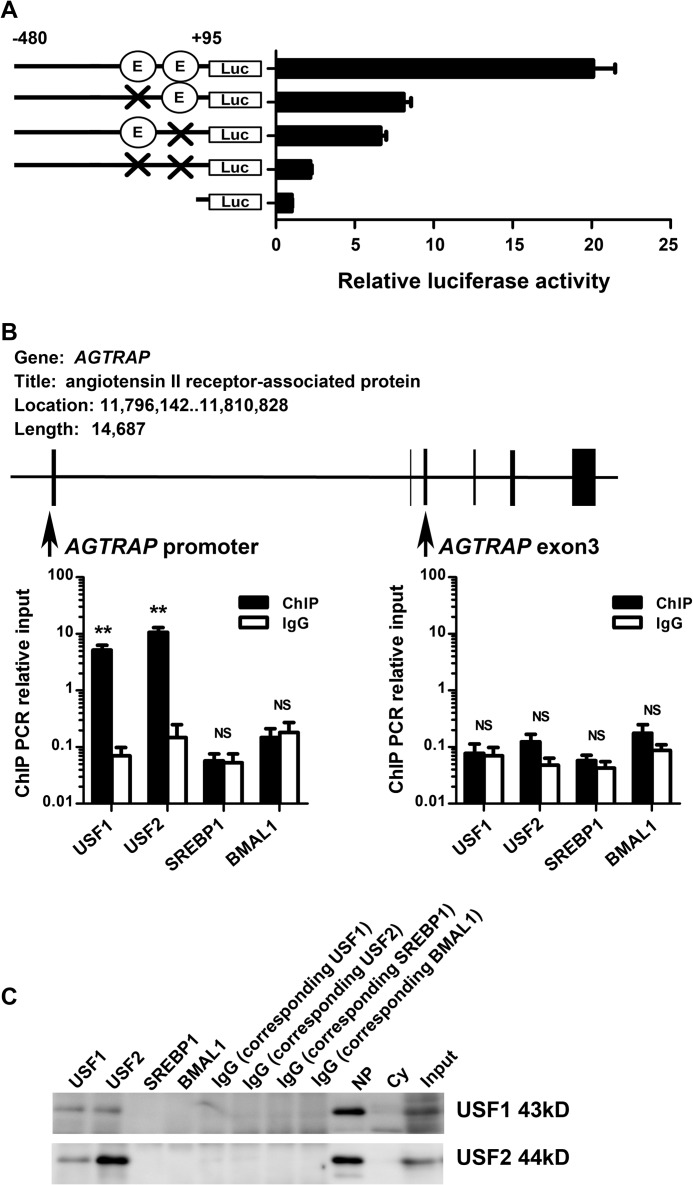
**Involvement of the E-box motifs in the regulation of the human *AGTRAP* promoter.** *A*, involvement of the two E-box motifs in the transcriptional activation of the *AGTRAP* promoter in HEK293 cells. The effects of mutations in the two E-box motifs on the transcriptional activity of the *AGTRAP* promoter-luciferase hybrid gene in HEK293 cells are shown. The relative luciferase activities were calculated relative to those achieved with the promoter-less control plasmid. Data are expressed as the means ± S.E. (*n* = 4). *B*, identification of USF1 and USF2 interaction with the *AGTRAP* proximal promoter region by ChIP analysis. Schematic representation of the *AGTRAP* gene structure and the approximate genomic positions for the enrichment of the *AGTRAP* promoter and exon3 region by ChIP assay are shown (*upper panel*). The results of ChIP assay with an anti-USF1 antibody, anti-USF2 antibody, anti-SREBP1 antibody, or anti-BMAL1 antibody are shown (*lower panel*). Data are expressed as the means ± S.E. (*n* = 4). **, *p* < 0.01, *versus* control IgG (rabbit anti-HA antibody). *NS,* not significant. *C*, identification of USF1 and USF2 in the co-immunoprecipitates from the *AGTRAP* proximal promoter region in ChIP analysis. Co-immunoprecipitated proteins with respective specific antibodies or their corresponding control IgG in ChIP assay were subjected to immunoblot analysis and were visualized by TrueBlot (Affymetrix). *NP,* nuclear extracts; *Cy,* cytosolic extracts; *Input,* input reference.

To evaluate the evolutionary and functional conservation of the regulation of *AGTRAP* gene expression by the E-box, we examined the activity of the *AGTRAP* proximal promoter with or without an E-box mutation using luciferase reporter assay. Because the promoter of the human isologous gene *AGTRAP* has two adjacently located E-box motifs (Fig. 2*A*), we analyzed both of them. As shown in Fig. 7*A*, the 575-bp human *AGTRAP* proximal promoter fragments (−480 to +95 of the putative transcriptional start site) exhibited substantial luciferase activity in human kidney-derived HEK293 cells. In addition, mutations of either of the two adjacently located E-box motifs significantly decreased the luciferase activity (Fig. 7*A*). Mutations of both E-box motifs further reduced the luciferase activity (Fig. 7*A*). These results indicate that the two adjacently located E-box motifs are important for the basal transcriptional activity directed by the *AGTRAP* promoter.

##### USF1 and USF2 Bind the AGTRAP Promoter Region

We further performed ChIP analysis to examine whether USF1, USF2, or BMAL1 physiologically interacts with the *AGTRAP* promoter region. For ChIP analysis, we prepared primer sets for the *AGTRAP* promoter region and internal exon 3 (Fig. 7*B*). A 161-bp fragment of the proximal upstream region of the two adjacently located E-box motifs in the *AGTRAP* promoter was recovered after immunoprecipitation of sheared genomic DNA from HEK293 cells with an anti-USF1 antibody and anti-USF2 antibody but not after immunoprecipitation with an anti-SREBP1 antibody or anti-BMAL1 antibody. Quantitative PCR analysis showed that USF1 and USF2 are present at the *AGTRAP* E-box promoter region, and the corresponding genomic DNA was enriched with an anti-USF1 antibody (**, *p* < 0.01, *versus* IgG control) and anti-USF2 antibody (**, *p* < 0.01, *versus* IgG control), respectively, but not with an anti-SREBP1 antibody or anti-BMAL1 antibody (Fig. 7*B*). Among these factors USF1 and USF2, but not BMAL1 and SREBP1, proteins were also detected in the co-immunoprecipitates from HEK293 cells on immunoblot analyses (Fig. 7*C*). However, USF1, USF2, SREBP1, and BMAL1 did not interact with the *AGTRAP* exon 3 region, which is a negative control region, without the E-box (Fig. 7*B*). These data indicate the occupancy by USF1 and USF2, but not SREBP1 or BMAL1, of the human *AGTRAP* promoter region.

## DISCUSSION

Despite the accumulating evidence supporting the involvement of an altered expression of *Agtrap* gene at local tissue sites in the pathogenesis of hypertension and related kidney injury, little is known about the transcriptional regulation of *Agtrap* expression. In this study, we showed that the promoter region from −150 to +72 of the mouse *Agtrap* 5′-flanking sequence, which is considered to contain important regulatory elements, directs *Agtrap* gene transcription in normal culture.

We analyzed the region from −381 to +72 based on the results showing maximum promoter activity. The results of luciferase assay using deletion mutants revealed the minimally required proximal promoter region from −150 to +72 that contains the X-box, E-box, and GC-box consensus motifs is able to direct substantial transcription of the *Agtrap* gene. Among these binding motifs, we confirmed that the E-box specifically binds Usf1 and Usf2 by employing EMSA, streptavidin-biotin complex assay, and ChIP. Such E-box-Usf1/Usf2 binding is functionally important in activating *Agtrap* expression for the following reasons: 1) mutation of the E-box to prevent Usf1/Usf2 binding reduces *Agtrap* promoter activity (Fig. 2); 2) transfection of siRNA for Usf1 increases and Usf2 decreases endogenous Agtrap mRNA and protein expression (Fig. 5), and 3) the decrease in Agtrap mRNA expression in the affected UUO kidney is accompanied by changes in Usf1 and Usf2 mRNA (Fig. 6). Taken together, these data indicate that Usf1 and Usf2 negatively and positively regulate *Agtrap* gene transcription, respectively. Because Usf1 and Usf2 bind to DNA with the same E-box sequence specificity, they most likely regulate *Agtrap* gene expression in a competitive manner.

Recently, the E-boxes in the promoter regions of renin and angiotensinogen were shown to be direct targets of Usf1 and Usf2 and suggested to be involved in the pathogenesis of both hypertension and renal injury (33, 56–58). In this study, it is shown that Agtrap, an emerging modulator of the renin-angiotensin system, is another target gene of Usf1 and Usf2, and that *Agtrap* gene expression is activated through the binding of Usf2 and inhibited through the binding of Usf1 to the same canonical E-box sequence in the *Agtrap* proximal promoter region.

Both Usf1 and Usf2 are reportedly activators of gene transcription via homodimerization or heterodimerization, with similar trans-activating capacities (59, 60), and they have also been proposed to function as repressors of a number of target genes (61). However, the results of this study show that Usf1 and Usf2 exert opposing regulatory effects on the expression of the same gene. Consistent with this notion, similarly opposing effects of Usf1 and Usf2 on the E-box of plasminogen activator inhibitor-1 gene, a key regulator of the fibrinolytic system, have been reported (62, 63). With respect to an interaction between Usf and other transcription factors, a previous study reported a contrasting functional and physical interaction between Usf and Sp1, a GC-box binding transcription factor, in the transcriptional regulation of the deoxycytidine kinase gene in liver-derived HepG2 cells (64). In the regulation of the deoxycytidine kinase promoter, the combination of Usf1 and Sp1 exhibited additive trans-activation at lower concentrations of Sp1, although Sp1 was inhibitory at higher levels, whereas trans-activation by Usf2 and Sp1 was synergistic in HepG2 cells (64). In this study, although the E-box and GC-box were found to be adjacently located in the *Agtrap* promoter, the results of luciferase assay showed a positive and independent stimulatory effect of these binding motifs in kidney-derived mDCT cells (Fig. 2), possibly because of a difference in the network of transcription factors in the liver and kidney. However, it is still possible that a functional interplay of Usf1 and Usf2 with putative transcription factors other than Sp1 is involved in the opposing regulatory effect exerted by Usf1 and Usf2 on *Agtrap* gene expression (Fig. 5). Further studies are needed to elucidate the molecular mechanisms, including kinase cascades, such as PI3K (28), which are involved in the differential regulatory functional effect of Usf1 and Usf2 on *Agtrap* gene expression. Studies are also needed to examine the possible role of E-box modulation by methylation at the core CpG in the Usf1/Usf2 recognition site (5′-CACpGTG-3′) in the regulation of the *Agtrap* promoter (64).

Cardiovascular and renal diseases are closely related to circadian rhythms, which are under the control of an internal biological clock mechanism. The binding of the transcription factors BMAL1 and CLOCK to multiple extra- and intragenic E-boxes is reported to play an important role in the circadian rhythm-related regulation of certain genes in peripheral, cardiovascular, and renal tissues (65–67). However, the present results do not indicate any significant interaction of BMAL1 with the E-box of the mouse *Agtrap* promoter (Fig. 4). This may be because the BMAL1-CLOCK heterodimer binds to multiple E-boxes of target genes despite there being a single E-box in the mouse *Agtrap* proximal promoter (66). The human *AGTRAP* promoter contains two adjacently located E-box motifs (Fig. 2). However, we did not obtain any evidence to indicate the interaction of BMAL1 with these two adjacently located E-box motifs in the *AGTRAP* promoter, at least in human kidney-derived cells (Fig. 7). Further studies are needed to examine the potential interaction of the BMAL1-CLOCK heterodimer with the adjacently located two E-box motifs in the *AGTRAP* promoter in other cells or tissues such as fat or liver, so as to exert cell type- or tissue-specific function. However, the results of the promoter assay and ChIP analysis clearly indicate the functional interactions of USF1/USF2 and the adjacently located two E-box motifs are involved in the regulation of the human *AGTRAP* promoter.

In summary, the results of this study show that Usf1 and Usf2 regulate *Agtrap* gene transcription through their interaction with the E-box in the mouse *Agtrap* promoter. Furthermore, the *in vitro* and *in vivo* results of siRNA transfection in mDCT cells and UUO in mice, respectively, suggest that Usf1 decreases and Usf2 increases *Agtrap* gene expression through the binding of Usf1/Usf2 to the E-box. We also demonstrated functional E-box-USF1/USF2 binding in the human *AGTRAP* promoter, thereby suggesting that a strategy of modulating the E-box-USF1/USF2 binding may have novel therapeutic potential.

## Footnotes

2 The abbreviations used are:

Ang II: angiotensin II
AT1R: angiotensin II type 1 receptor
ATRAP/Agtrap: angiotensin II type 1 receptor-associated protein
mDCT: mouse distal convoluted tubule
USF/Usf: upstream stimulatory factor
UUO: unilateral ureteral obstruction
ODN: oligonucleotide.
